# The association of neutrophil to lymphocyte ratio, mean platelet volume, and platelet distribution width with diabetic retinopathy and nephropathy: a meta-analysis

**DOI:** 10.1042/BSR20180172

**Published:** 2018-06-27

**Authors:** Juxiang Liu, Xingguang Liu, Youpeng Li, Jinxing Quan, Suhong Wei, Siqin An, Ruifei Yang, Jing Liu

**Affiliations:** 1 The First Clinical Medical College of Lanzhou University, Lanzhou 73000, Gansu, China; 2 Department of Endocrinology, Gansu Provincial People’s Hospital, Lanzhou, China; 3 Department of Cardiac Surgery, Gansu Provincial People’s Hospital, Lanzhou, China; 4 Department of Gastroenterology, Minqin County People’s Hospital in Gansu Province, Gansu, China; 5 Department of Endocrinology, Key Laboratory of Endocrine and Metabolic Diseases of Gansu Province, Lanzhou, China

**Keywords:** diabetic nephropathy, diabetic retinopathy, mean platelet volume, Neutrophil to lymphocyte ratio, platelet distribution width

## Abstract

The aim of the present study was to investigate the correlation of neutrophil to lymphocyte ratio (NLR), mean platelet volume (MPV), and platelet distribution width (PDW) with diabetic nephropathy (DN) and diabetic retinopathy (DR). We searched for eligible studies from PubMed, Embase, Web of Science, and CNKI up to 1 December 2017. Standardized mean difference (SMD) was calculated with a confidence interval (CI) of 95%. A total of 48 studies were included in our meta-analysis. Compared with patients with type Ⅱ diabetes mellitus (T2DM) and without DR, NLR, MPV, and PDW were higher in patients with DR (SMD = 0.77; 95% CI: 0.49–1.05; *P*<0.001; SMD = 0.68; 95% CI: 0.36–0.99; *P*<0.001; SMD = 0.52; 95% CI: 0.28–0.76; *P*<0.01). Compared with patients with T2DM and without DN, NLR, MPV, and PDW were higher in patients with DN (SMD = 0.63; 95% CI: 0.43–0.83; *P*<0.001; SMD = 0.81; 95% CI: 0.36–1.25; *P*<0.001; SMD = 0.70; 95% CI: 0.50–0.90; *P*<0.001). We also found that MPV was strongly associated with the severity of DR, and NLR was closely related to the degree of DN. Our findings indicated that NLR, MPV, and PDW could be recommended as inexpensive diagnostic biomarkers for DN and DR. However, considering several limitations in the present study, further high-quality clinical studies should be performed to investigate the relationship of NLR, MPV, and PDW to DN and DR.

## Introduction

Diabetes is a global health burden and its morbidity and mortality are increasing in recent decades, especially in populations from the low- and middle-income countries [1,2]. Diabetes is a systemic metabolic disorder disease that can result in several long-term micro- and macrovascular complications, including diabetic retinopathy (DR) and diabetic nephropathy (DN). It was reported that ~28.5–40.3% of patients with type II diabetes mellitus (T2DM) suffered from DR and 25–40% had DN [3–5]. To make things worse, DR and DN are serious microvascular complications and have been considered as the major cause of blindness and end-stage renal failure, respectively [3–5]. Thus, it is very significant for clinicians and researchers to elucidate pathogenesis of DR and DN and identify high-risk patients.

Diabetes mellitus (DM) is a chronic systemic disease. A flood of literatures have suggested that chronic inflammation was involved in insulin resistance and DM development [6–9]. Moreover, accumulated evidence have implicated that inflammatory response also plays a key part in the development and progression of DR and DN [10–13]. In the studies exploring the association of chronic inflammation with DR and DN, elevated C-reactive protein levels were observed in patients with DR, as compared in DM patients without DR. Besides, various inflammatory cytokines, such as interleukin-1, -6, -8, and tumor necrosis factor α were also considered to be tightly associated with the pathogenesis of DN. In recent years, neutrophil to lymphocyte ratio (NLR) as a novel indicator of systemic inflammatory response in various diseases emerged and was considered as an independent predictor of major morbidity, mortality, and long-term survival in many clinical settings [14–16]. Particularly, an increasing evidence showed that NLR was also closely related to the development and progression of DR and DN [13,17,18]. In addition to inflammation, prothrombotic state in DM patients was also reported to be implicated in the development and acceleration. Mean platelet volume (MPV), which can be readily obtained from the hematological analyzers, is a parameter to evaluate the platelet size and can reflect the platelet functions and activation. Platelets with higher MPV value have more metabolic and enzymatic activities than lower size, with more thromboxan-A_2_, b-thromboglobulin, and adhesion molecules releasing [19,20]. Besides, platelet distribution width (PDW) is a parameter that can directly mirror the variability of platelet size. Its high value indicates increased production of larger reticulated platelet and also suggests high activity. Thus, numerous studies have focussed on investigating the relationship of MPV and PDW to the development and progression of DR and DN, and several studies suggested that there was a positive link.

However, in retrospect, the conclusions regarding the relationship of NLR, MPV, and PDW to the development and progression of DR and DN were conflicting [21–23]. One of the reasons for the inconsistency might be that the sample size was small in a single study and the power of statistical results was challenged. Therefore, we herein performed a meta-analysis and systematic review to comprehensively assess the correlation of NLR, MPV, and PDW with DR and DN.

## Methods

This meta-analysis was conducted according to the Preferred Reporting Items for Systematic Reviews and Meta-Analyses (PRISMA) statements [24]. Study selection, data extraction, and quality assessment were completed by two investigators separately.

### Study selection

We searched PubMed, Embase, Web of Science, and CNKI up to 11 December 2017 to retrieve eligible studies. Literature search was performed using the following keywords: ‘neutrophil to lymphocyte ratio/NLR’ or ‘mean platelet volume/MPV’ or ‘platelet distribution/PDW’ in combination with ‘diabetic retinopathy/DR’ or ‘diabetic nephropathy/DN’. Moreover, references of the included studies were also examined. No language or date limitations were applied in this meta-analysis.

### Inclusion criteria

Studies that met the following inclusion criteria were included in this meta-analysis. (i) The enrolled patients in studies were clearly diagnosed with T2DM. (ii) The data of NLR, MPV, and PDW were presented as the mean value with sufficient data (SD). (iii) There were comparisons of NLR, MPV, and PDW values between DM, and DR or DN.

### Exclusion criteria

Studies have been excluded based on the following criteria: (i) reviews, editorials, meeting abstracts, case reports, and non-comparative studies; (ii) duplicate publications; (iii) data were unavailable or only reported in abstracts; (iv) articles were submitted by same authors or institution which might have overlapping patients.

### Data extraction

All the data were extracted from each eligible article by two independent reviewers, and all disagreements on eligibility during the extraction were discussed and resolved through a consensus. The extracted information included the name of first author, publication year, study design, country, number of patients in case and control groups, mean age and sex in each group, the mean value with SD of NLR, MPV, PDW.

### Methodology quality assessment

The Newcastle–Ottawa scale (NOS) was used for the assessment of included studies [25]. And a score of 0–9 points has been assigned to each study, and studies ≥6 points were considered to be of relatively high quality.

### Statistical analysis

The statistical analysis was performed by using STATA version 12.0 (Stata Corporation, College Station, TX, U.S.A.). Standard mean difference (SMD) and its 95% confidence interval (CI) was used to depict the pooled continuous variables. If the data of NLR, MPV, and PDW were reported in non-proliferative DR/proliferative DR (NPDR/PDR) or microalbuminuria/macroalbuminuria (MIA/MA) groups, the data in NPDR or MIA were used to compare with control group. Based on the 95% CI not crossing 0, the SMD < 0 suggested that the specific hematologic inflammatory marker increased in control group. In addition, statistical heterogeneity amongst the studies was evaluated by Cochrane *Q* test (χ^2^) and *I^2^* test. Moreover, a random-effect model was conducted to calculate the SMD when significant heterogeneity existed amongst some studies (*P*<0.05 and *I^2^* ≥ 50%), otherwise, fixed-effect model was used. Sensitivity analysis was performed to verify the stabilization of our results, by excluding the articles one by one in each corresponding group, and then obtained meta-analysis results from the remaining studies. Furthermore, the Begg’s test and Egger’s test were performed to evaluate the publication bias [26,27]. If the significant publications bias exist (*P*<0.01 for Begg’s test and Egger’s test), Duval wnd Tweedie’s [28] non-parametric trim-and-fill method was conducted to evaluate the effects of bias on the results.

## Results

### Study selection

**Figure 1:**
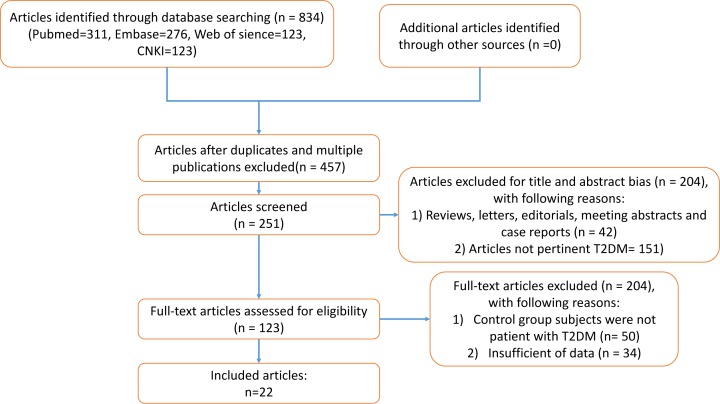
Flow chart of study selection process

The flow chart diagram of the process of study selection was shown in Figure 1. A total of 190 articles were yielded through primary study searching for PubMed, Embase, Web of Science, and CNKI. Of these studies, 44 were from PubMed, 67 from Embase, 52 from Web of Science, and 27 from CNKI. Amongst these studies, 17 articles were removed because of duplication, and 105 publications were excluded due to reviews, meeting abstracts, and studies not pertinent to DR or DN after screening the title and abstract. Thus, the remaining 68 articles were left for the full-text review. Of the 68 studies, 9 articles were excluded since control group’s subjects were not DM patients, but healthy people, and 11 articles were removed owing to the lack of available data. Finally, 48 articles with 9118 patients were enrolled in this meta-analysis [21–23,29–73].

### Characteristics of included studies

All the selected studies were published from 2000 to 2017. The number of the included subjects in the case group and the control group ranged from 20 to 328 and 20 to 192, respectively. Of these 48 articles, 27 came from China [22,30,34,36,37,41,43–46,48,49,55–60,64,65,67,68,70–73], 16 from Turkey [21,23,29,31,35,38,40,42,47,50–54,61,69], 3 from India [32,63,39], 1 from Greece [62] and 1 from Kazakhstan [33]. Amongst these studies, 21 reported NLR (9 enrolled patients with DR, 12 enrolled patients with DN) [21,23,31,32,34,35,38,41–43,45,47,50–54,56–60,62,63,65,67–69,71–73], 33 reported the data of MPV (23 enrolled patients with DR, 10 enrolled patients with DN) [22,29,30,33,36–40,44,46,48–50,55,61,64,66,70,73], and 12 reported PDW (12 enrolled patients with DR, 3 enrolled patients with DN) [21,23,32,41,43,50,58–60,65,67–69,71]. The basic characteristics and data of our interest of the eligible studies were summarized in Tables 1–6. Moreover, the methodological quality of the included articles were assessed according to NOS, and the scores ranged from 5 to 7, indicating that the quality of selected studies were moderate to high.

**Table 1:** The main characteristics of the included studies on the relationship of MPV and PDW with diabetic nephropathy

			No. of patients					
Author Year	Study design	Country	Proteinuria (−)	Proteinuria (+)		Age Mean/Median	Male/Female	NOS
				MIA	MA			
Bavbek 2007 [31]	R	Turkey	39	75		P(-): 59.5 ± 8.1 P(+): 59.4 ± 10.8	P(-): 27/12 P(+): 47/28	6
Buch 2017 [32]	R	India	264	36		NR	NR	7
Dindar 2013 [35]	R	Turkey	35	25		NR	NR	6
Kahraman 2016 [39]	R	Turkey	54	34	24	P(-): 59.9 ±7.9 MIA: 64.1 ±8.9 MA: 56.8 ±12.5	P(-): 16/38 MIA: 16/18 MA: 7/17	7
UNUBOL 2011 [42]	R	Turkey	230	124		P(-): 56.87±9.65 P(+): 55.73±11.07	P(-): 108/122 P(+): 54/70	7
Turgutalp 2012 [47]	R	Turkey	160	144		P(-): 55.14 + 10.90 P(+): 54.08 + 12.16	P(-): 77/83 P(+): 74/70	7
Cui 2006 [34]	R	China	30	42		NR	P(-): 15/15 P(+): 20/22	6
Liang 2008 [41]	R	China	68	68		P(-): 69.1 P(+): 67.1	P(-): 32/36 P(+): 30/38	6
Ma 2007 [43]	R	China	72	80		P(-): 67.6 P(+): 68.3	P(-): 32/40 P(+): 34/46	6
Sun 2007 [45]	R	China	35	31	30	P(-): 57.3±14.6 MIA: 57.6±13.5 MA: 58.2±13.2	P(-): 18/17 MIA: 16/15 MA: 16/14	6
Akbas 2014 [29]	R	Turkey	132	34	34	P(-): 56.03±10.54 MIA: 60.35±11.40 MA: 59.03±9.61	P(-): 69/63 MIA: 16/18 MA: 18/16	7
Ao 2017 [30]	R	China	80	100	100	P(-): 57.1±11.1 MIA: 56.4±8.5 MA: 70.6±7.6	P(-): 41/39 MIA: 55/45 MA: 56/44	7
Ciray 2015 [33]	R	USA	52	62		P(-): 56.9 ± 10.1 P(+): 62.1 ± 11.7	NR	6
Demirtas 2015 [53]	R	Turkey	193	114		NR	NR	6
Huang 2014 [37]	R	China	138	115		NR	NR	6
Feng 2016 [36]	R	China	38	52	30	P(-): 61.8±9.1 MIA: 57.7±10.80 MA: 63.1±10.1	P(-): 23/15 MIA: 18/34 MA: 16/14	6
Kahraman 2016 [38]	R	Turkey	54	34	24	P(-): 59.9 ±7.9 MIA: 64.1 ±8.9 MA: 56.8 ±12.5	P(-): 16/38 MIA: 16/18 MA: 7/17	7
Khandare 2017 [39]	R	India	56	59		P(-): 50.05±11.29 P(+): 52.29±11.45	P(-): 26/33 P(+): 25/31	5
Ren 2015 [44]	R	China	165	124	41	P(-): 56.90±11.25 MIA: 55.35±11.26 MA: 55.02±10.04	P(-): 81/84 MIA: 71/53 MA: 26/15	6
Tian 2017 [46]	R	China	72	49		P(-): 57.32±10.43 P(+): 61.14±12.03	P(-): 39/33 P(+): 29/20	5
Xu 2016 [48]	R	China	86	74		P(-): 44.41±10.81 P(+): 49.22±12.71	P(-): 48/38 P(+): 48/26	6
Zhu 2016 [49]	R	China	180	112		P(-): 45.270±7.542 P(+): 48.070±9.377	P(-): 80/100 P(+): 60/52	7

Abbreviation: R: Retrospective study design; P: Proteinuria; MIA: Microalbuminuria; MA: Macroalbuminuria

**Table 2:** values of NLR in T2DM subjects with and without diabetic nephropathy

	Neutrophil to Lymphocyte Ratio					
First author	Proteinuria (−)		Proteinuria (+)			
			MIA		MA	
	Mean	SD	Mean	SD	Mean	SD
Akbas 2014 [29]	1.56	0.85	1.96	0.73	2.03	1.52
Ao 2017 [30]	1.67	0.54	1.85	0.77	2.44	0.73
Ciray 2015 [33]	1.77	0.56	2.27 ± 1.25			
Demirtas 2015 [53]	1.68	0.20	1.99±0.28			
Huang 2014 [37]	2.20	0.62	2.48±0.59			
Feng 2016 [36]	2.03	1.26	2.72	1.47	3.54	1.15
Kahraman 2016 [38]	1.9	0.9	2.6	1.0	3.6	1.3
Khandare 2017 [39]	1.94	0.65	2.83±0.85			
Ren 2015 [44]	1.84	0.61	2.14	1.30	2.74	1.74
Tian 2017 [46]	1.76	0.76	2.14±1.06			
Xu 2016 [48]	1.81	0.56	2.27±0.92			
Zhu 2016 [49]	1.836	0.733	2.654±1.465			

**Table 3:** The values of mean platelet volume and platelet distribution width T2DM subjects with and without diabetic nephropathy

	Mean Platelet Volume (fl)						Platelet Distribution Width (%)					
First author	Proteinuria (−)		Proteinuria (+)				Proteinuria (−)		Proteinuria (+)			
			MIA		MA				MIA		MA	
	Mean	SD	Mean	SD	Mean	SD	Mean	SD	Mean	SD	Mean	SD
Bavbek 2007 [31]	8.3	1.3	8.6±1.3				NR	NR	NR	NR	NR	
Buch 2017 [32]	10.47	2.06	11.01±2.23				13.99	3.48	15.72±3.97			
Dindar 2013 [35]	10.80	1.09	11.07±1.13				NR	NR	NR			
Kahraman 2016 [38]	8.8	1.0	8.8	1.2	8.3	0.9	NR	NR	NR			
UNUBOL 2011 [42]	8.61	1.06	8.95±1.14				NR	NR	NR			
Turgutalp 2012 [47]	9.85	0.74	10.61± 0.55				NR	NR	NR			
Cui 2006 [34]	8.3	1.1	9.1±1.3				NR	NR	NR			
Liang 2008 [41]	10.2	1.2	13.2±1.3				17.1	1.2	18.2±1.2			
Ma 2007 [43]	8.3	1.1	10.3±1.3				17.2	1.3	18.1±1.3			
Sun 2007 [45]	8.25	1.13	10.13	2.01	11.19	2.56	NR	NR	NR			

Abbreviation: MIA: Microalbuminuria; MA: Macroalbuminuria

**Table 4:** The main characteristics of the included studies on the relationship of MPV and PDW diabetic retinopathy

			No. of					
Author Year	Study design	Country	Cases with DM	Cases with NPDR	Cases with PDR	Age Mean/Median	Male/Female	NOS
Akdoğan 2016 [50]	Retrospective	Turkey	158	120		DM:57.3±12.2 DR: 59.8±9.2	DM: 59/99 DR: 47/73	7
Ateş 2009 [51]	Retrospective	Turkey	30	30	30	NR	NR	5
Buch, 2017 [32]	Retrospective	India	220	80		NR	NR	6
Citirik 2015 [23]	Prospective	Turkey	43	45	52	DM:60.4 ± 8.5 NPDR:61.4 ± 9.3 PDR:59.4 ± 7.2	DM: 22/21 NPDR: 17/28 PDR: 20/32	6
Demirtas 2015 [53]	Prospective	Turkey	240	67		NR	NR	7
Dindar 2013 [35]	Retrospective	Turkey	36	24		NR	NR	6
GÜNGÖR 2016 [54]	Retrospective	Turkey	50	52		NR	DM: 19/31 DR: 18/34	6
Li 2014 [56]	Retrospective	China	72	67	70	DM:54.2 ± 9.2 NPDR:57.7± 10.0 PDR:58.3 ± 9.4	DM:35/37 NPDR:34/33 PDR:33/37	7
Li 2013 [57]	Retrospective	China	103	132		NR	NR	7
Li 2016 [58]	Retrospective	China	52	47		DM: 55.1±15.2 DR: 54.1±10.8	DM: 31/21 DR:26/21	6
Ma 2017 [59]	Retrospective	China	20	20	20	DM:57.3± 6.5 NPDR:60.8± 7.3 PDR:57.6 ± 7.3	DM:12/8 NPDR:8/12 PDR:12/8	5
Niu 2013 [60]	Retrospective	China	20	25		DM:46.5±8.3 DR:51.2±8.3	DM:12/8 DR:13/12	6
Papanas 2004 [62]	Retrospective	Greece	89	167		NR	NR	7
Radha 2016 [63]	Retrospective	India	30	14		NR	NR	5
Sheng 2017 [64]	Retrospective	China	102	102		NR	NR	7
Tetikoğlu 2016 [21]	Retrospective	Turkey	63	56	80	NR	NR	6
Tuzcu 2014 [52]	Retrospective	Turkey	70	64	58	DM: 55.8 ±10.5 NPDR: 60.1 ± 8.6 PDR: 57.5 ± 9.3	DM:38/32 NPDR:32/32 PDR:31/27	6
Wei 2017 [67]	Retrospective	China	94	52	40	DM: 58.14 ± 11.93 DR: 58.42 ± 12.09	DM:50/44 DR:49/43	7
Xu 2014 [68]	Retrospective	China	45	40		NR	DM: 23/22 DR:26/14	6
Yilmaz 2016 [69]	prospective	Turkey	89	88	86	DM: 60.9 ± 6.3 NPDR: 62.7 ± 7.2 PDR: 61.7 ± 7.9	DM:49/40 NPDR:48/40 PDR:49/37	7
Yu 2000 [71]	Retrospective	China	60	40		NR	NR	6
Yue 2015 [22]	Retrospective	China	125	62	59	DM: 56.00 ± 3.75 NPDR: 53.50 ± 3.56 PDR: 56.0 ± 3	DM:73/52 NPDR:34/28 PDR:28/31	6
Zhang 2002 [72]	Retrospective	China	20	20		DM:58.0±9.0 DR:60.0±1.3	DM: 9/11 DR:8/12	5
Zhou 2016 [73]	Retrospective	China	328	51		DM: 57 ±16 DR: 63 ±15	DM: 198/130 DR: 34/17	6
Akdoğan 2016 [50]	Retrospective	Turkey	158	120		DM:57.3±12.2 DR: 59.8±9.2	DM: 59/99 DR: 47/73	7
Ciray 2015 [33]	Retrospective	Kazakhstan	59	55		DM: 57.8 ± 11.5 DR: 61.8 ± 10.8	NR	6
Kuang 2015 [55]	Retrospective	China	62	44	22	DM:60.73 ± 11.24 NPDR:60.50 ± 8.45 PDR:55.18 ± 13.05	DM:29/33 NPDR:17/27 PDR:11/11	6
Öztürk 2013 [61]	Retrospective	Turkey	97	79	NR	NR	NR	6
Shen 2016 [64]	Retrospective	China	118	134	58	DM:55.19 ± 5.51 NPDR:58.04 ± 7.53 PDR:59.84 ± 8.76	DM:63/55 NPDR:73/61 PDR:34/24	6
Ulu 2013 [66]	Retrospective	Turkey	34	24		NR	NR	6
Wei 2017 [67]	Retrospective	China	94	52	40	DM: 58.14 ± 11.93 DR: 58.42 ± 12.09	DM:50/44 DR:49/43	6
Wang 2015 [18]	Retrospective	China	138	131		DM: 60.3 ± 6.0 DR: 66.6 ± 5.8	DM:65/73 DR:53/78	6
Yin 2015 [70]	Retrospective	China	64	28	36	DM: 56.83 ± 9.01 NPDR: 53.09 ± 8.82 PDR: 53.16 ± 10.64	DM:35/29 NPDR:13/15 PDR:19/17	6
Yue 2015 [22]	Retrospective	China	125	62	59	DM: 56.00 ± 3.75 NPDR: 53.50 ± 3.56 PDR: 56.0 ± 3	DM:73/52 NPDR:34/28 PDR:28/31	6
Zhou 2016 [73]	Retrospective	China	328	51		DM: 57 ±16 DR: 63 ±15	DM: 198/130 DR: 34/17	6

Abbreviation: DM: Diabetes mellitus; NPDR: Non-proliferative diabetic retinopathy; PDR: Proliferative diabetic retinopathy.

**Table 5:** values of NLR in T2DM subjects with and without diabetic retinopathy

	Neutrophil to Lymphocyte Ratio (%)						Platelet to Lymphocyte Ratio (%)					
First author	Cases without DR		Cases with NPDR		Cases with PDR		Cases without DR		Cases with NPDR		Cases with PDR	
	Mean	SD	Mean	SD	Mean	SD	Mean	SD	Mean	SD	Mean	SD
Akdoğan [50]	2.4	1.9	3.0 ± 4.4				116	66	140 ± 87			
Ciray 2015 [33]	1.99	1.03	2.10 ± 1.02				NR	NR	NR		NR	
Kuang 2015 [55]	1.68	0.48	2.20	0.40	2.58	0.41	NR	NR	NR		NR	
Öztürk 2013 [61]	2.04	0.72	2.58±1.34				NR	NR	NR		NR	
Shen 2016 [64]	1.52	0.26	1.68	0.21	1.95	0.17	NR	NR	NR		NR	
Ulu 2013 [66]	1.96	0.86	3.59 ±2.07				NR	NR	NR		NR	
Wei 2017 [67]	NR	NR	NR	NR	NR		98.46	10.63	127.25 ± 12.98			
Wang 2015 []	2.1	1.3	3.7 ± 1.4				NR	NR	NR		NR	
Yin 2015 [70]	1.54	0.55	1.83	0.59	2.15	0.77	NR	NR	NR		NR	
Yue 2015 [22]	1.74	0.245	2.05	0.3	1.91	0.28	94.04	12.365	105.07	17.47	115.73	14.54
Zhou 2016 [73]	2.4	1.5	4.4±2.7				NR	NR	NR		NR	

Abbreviation: NLR: Neutrophil to lymphocyte ratio; T2DM: Type 2 diabetes mellitus; NPDR: Non-proliferative diabetic retinopathy; PDR: Proliferative diabetic retinopathy.

**Table 6:** values of MPV and PDW in T2DM subjects with and without diabetic retinopathy

	Mean Platelet Volume (fl)						Platelet Distribution Width (%)					
First author	Cases with DM		Cases with NPDR		Cases with PDR		Cases with DM		Cases with NPDR		Cases with PDR	
	Mean	SD	Mean	SD	Mean	SD	Mean	SD	Mean	SD	Mean	SD
Akdoğan 2016 [50]	9.7	1.2	9.6 ± 1.0				16.2	0.8	16.2 ± 0.5			
Ateş 2009 [51]	7.76	0.72	7.94	0.61	8.18	0.89	NR	NR	NR	NR	NR	NR
Buch, 2017 [32]	10.24	2.04	11.40 ± 1.96				13.94	3.33	14.92 ± 4.14			
Citirik 2015 [23]	7.94	0.63	8.05	0.76	8.10	0.68	14.85	1.27	15.15	1.19	14.92	1.15
Demirtas 2015 [53]	9.20	0.92	9.54±0.88				NR	NR	NR	NR	NR	NR
Dindar 2013 [35]	10.68	1.68	11.26 ± 1.08				NR	NR	NR	NR	NR	NR
GÜNGÖR 2016 [54]	8.8	1.1	9.3 ± 1.0				NR	NR	NR	NR	NR	NR
Li 2014 [56]	7.8	1.1	8.3	1.38	8.9	1.65	NR	NR	NR	NR	NR	NR
Li 2013 [57]	9.05	0.44	9.73 ± 0.53				NR	NR	NR	NR	NR	NR
Li 2016 [58]	10.39	0.90	10.72 ± 1.57				13.80	3.32	16.17 ± 1.66			
Ma 2017 [59]	8.12	0.82	8.96	0.86	10.76	1.12	15.66	2.37	17.85	2.26	17.90	2.41
Niu 2013 [60]	10.25	2.04	14.21 ± 2.35				16.05	1.56	18.12 ± 1.25			
Papanas 2004 [62]	10.9	1.1	15.8 ± 1.3				NR	NR	NR	NR	NR	NR
Radha 2016 [63]	8.39	0.67	9.2 ± 0.61				NR	NR	NR	NR	NR	NR
Sheng 2017 [65]	9.76	0.86	10.17 ± 0.92				11.31	1.67	12.04 ± 1.88			
Tetikoğlu 2016 [21]	8.51	1.0	8.42	0.9	8.91	0.7	16.9	0.7	16.8	0.7	17.3	3.1
Tuzcu 2014 [52]	7.90	1.26	8.20	1.55	8.78	1.73	NR	NR	NR	NR	NR	NR
Wei 2017 [67]	11.12	1.3	11.50	1.39	11.56	1.06	13.70	2.90	14.40	2.88	14.20	1.99
Xu 2012 [68]	11.21	1.71	13.44 ± 2.01				15.98	1.23	17.41 ± 1.42			
Yilmaz 2016 [69]	7.84	0.76	7.90	0.85	8.31	0.76	13.02	1.29	13.49	1.18	13.77	1.26
Yu 2000 [71]	10.93	2.35	13.08 ± 2.04				17.77	1.97	21.48 ± 5.94			
Yue 2015 [22]	NR	NR	NR				NR	NR	NR			
Zhang 2002 [72]	9.82	1.53	10.19 ± 2.06				NR	NR	NR	NR	NR	NR
Zhou 2016 [73]	10.0	1.1	10.4 ± 1.1				NR	NR	NR	NR	NR	NR

Abbreviation: T2DM: Type 2 diabetes mellitus; NPDR: Non-proliferative diabetic retinopathy; PDR: Proliferative diabetic retinopathy.

### Meta-analysis results

#### The association of NLR with DN and degree of albuminuria

**Figure 2:**
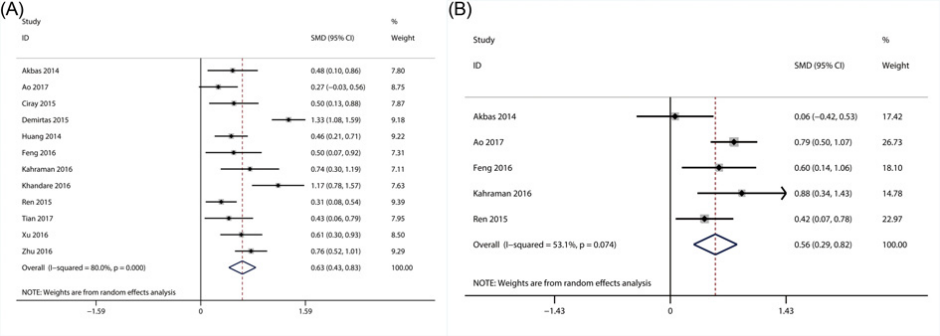
Results of forest plot of the relationship between NLR and DN Forest plot of the relationship between NLR and DN (**A**); forest plot of the relationship between NLR and the degree of DN (**B**).

A total of 12 studies involving 2404 patients were included for the meta-analysis of the association of NLR with the presence of DN [29,30,33,36–40,44,46,48,49], and 5 articles reported the data regarding the relationship between NLR and degree of albuminuria (MIA and MA) [29,30,36,38,44]. Random-effect model was applied since the significant heterogeneity was present for the presence of DN (*I^2^* = 80.0; *P*<0.01), and degree of albuminuria (*I^2^* = 53.1; *P*=0.07). From the results of our meta-analysis, NLR level was significantly higher in patients with DN (SMD = 0.63; 95% CI: 0.43–0.83) (Figure 2A) and MA (SMD = 0.56; 95% CI: 0.29–0.82) (Figure 2B), as compared with those without DN and MIA.

#### The association of NLR with DR

**Figure 3:**
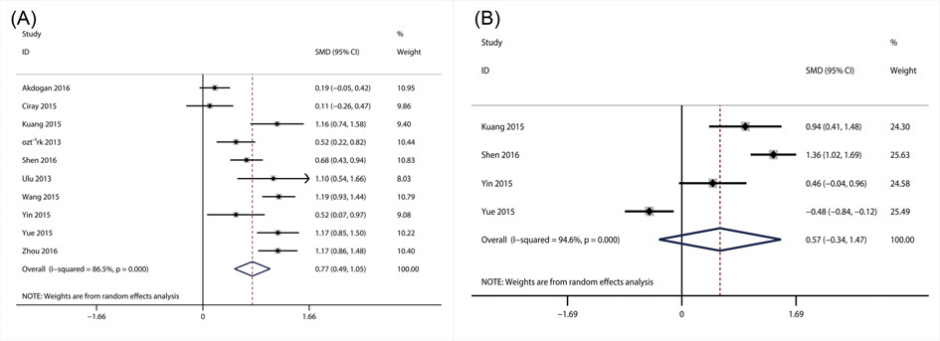
Results of forest plot of the relationship between NLR and DR Forest plot of the relationship between NLR and DR (**A**); forest plot of the relationship between NLR and the degree of DR (**B**).

A total of ten studies with 1911 patients, which provided the data about the association of NLR with the presence of DR [22,33,50,55,61,64,66,70,73], were included in this meta-analysis. The heterogeneity tests showed the extinction of significant heterogeneity (*I^2^* = 86.5; *P*<0.01), so random-effect model was used. The results suggested that NLR level was substantially higher in patients with DR (SMD = 0.77; 95% CI: 0.49–1.05) (Figure 3A) than those without DR. Additionally, four articles with 443 patients were also included for the meta-analysis of the association between NLR and the degree of DR (NPDR and PDR). However, no significant association between NLR and the degree of DR was observed in this meta-analysis (SMD = 0.57; 95% CI: −0.34–1.47) (Figure 3B).

#### The association of MPV with DN

**Figure 4:**
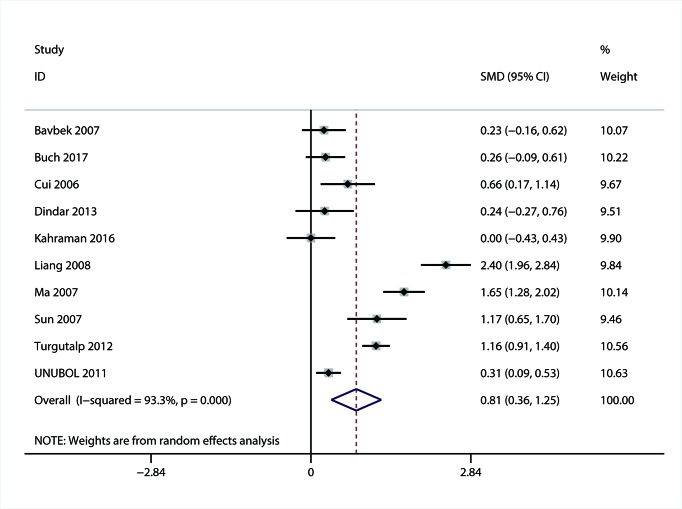
Forest plot of the relationship between MPV and DN

A total of ten studies with 1700 patients reported the data of MPV for DN [31,32,34,35,38,41–43,45,47]. Considering the significant heterogeneity (*I^2^* = 93.3; *P*<0.01), random-effect meta-analysis was conducted and the results showed that MPV was higher in patients with DN than DM patients without DN (SMD = 0.81; 95% CI: 0.36–1.25) (Figure 4).

#### The association of MPV with DR

**Figure 5:**
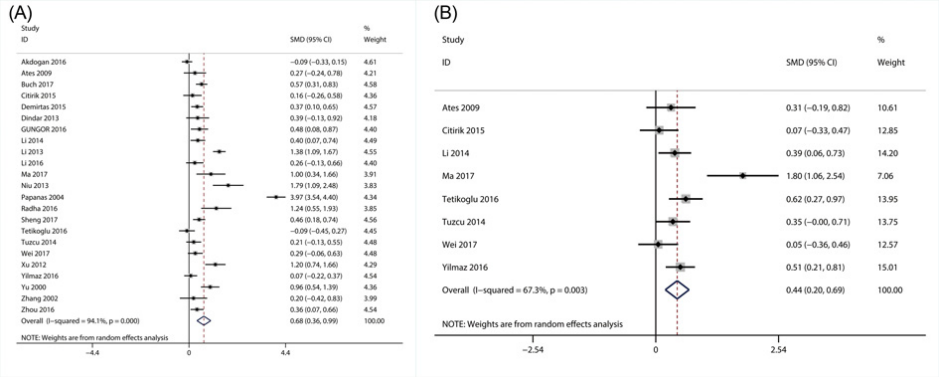
Results of forest plot of the relationship between MPV and DR Forest plot of the relationship between MPV and DR (**A**); forest plot of the relationship between MPV and the degree of DR (**B**).

There were 23 articles with 3437 patients, which had data of MPV for DR [21,23,32,35,50–54,56–60,62,63,65,67–69,71–73]. Random-effects model was used to pool the data because of the significant heterogeneity amongst the included studies (*I^*2*^* = 94.1; *P*<0.01). From the result, we observed that MPV was higher in DR group as compared with DM patients without DR (SMD = 0.68; 95% CI: 0.36–0.99) (Figure 5A). Moreover, a total of eight studies with 858 patients investigated the association of MPV with the degree of DR. Similarly, random-effect model was applied due to the significant heterogeneity (*I^2^* = 67.3; *P*<0.01). The result showed that MPV level was significantly higher in patients with PDR than in NPDR (SMD = 0.44; 95% CI: 0.20–0.69), indicating that MPV was closely correlated with the progression of DR (Figure 5B).

#### The association of PDW with DN

**Figure 6:**
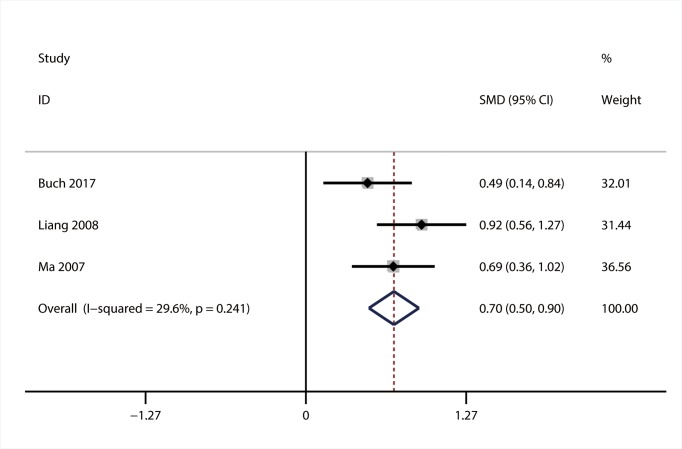
Forest plot of the relationship between PDW and DN

Three articles with 588 patients were included in the meta-analysis of the association between PDW and DN [32,41,43]. Heterogeneity analysis indicated the absence of significant heterogeneity (*I^2^* = 29.6; *P*=0.241), so fixed-effect model was used. The result of meta-analysis showed that PDW level was elevated in DR group compared with DM group (SMD = 0.70; 95% CI: 0.50–0.90) (Figure 6).

#### The association of PDW with DR

**Figure 7:**
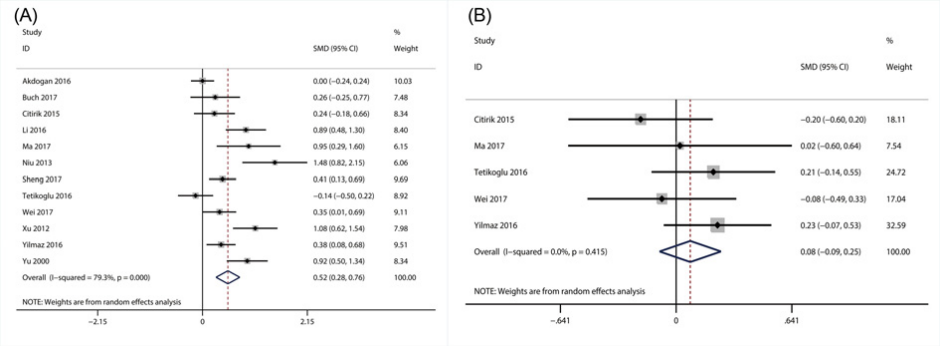
Results of forest plot of the relationship between PDW and DR Forest plot of the relationship between PDW and DR (**A**); forest plot of the relationship between PDW and the degree of DR (**B**).

A total of 12 articles with 1681 patients explored the relationship between PDW and DR [21,23,32,50,58–60,65,67–69,71]. Considering the significant heterogeneity amongst the 12 studies (*I^2^* = 79.3; *P*<0.01), random-effect model was applied when we performed the meta-analysis. As the results showed, PDW level was significantly higher in DR group (SMD = 0.52; 95% CI: 0.28–0.76) than in DM group (Figure 7A). Furthermore, there were five studies with 539 patients, which referred to the relationship between PDW and the severity of DR. Hence, in the present study, we also performed the meta-analysis to investigate relationship between PDW and the severity of DR. However, the result indicated that there was no significant association of PDW with the severity of DR (SMD = 0.08; 95% CI: –0.09–0.25) (Figure 7B).

### Sensitive analysis

**Figure 8:**
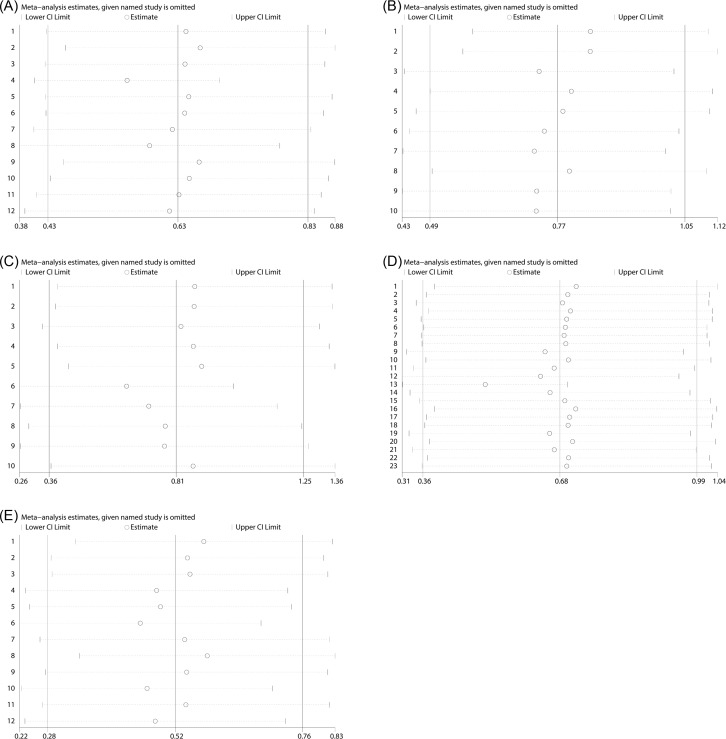
The sensitive analysis of selected studies NLR for DN (**A**), NLR for DR (**B**), MPV for DN (**C**), MPV for DR (**D**), PDW for DR (**E**).

For the purpose of verifying the robustness of the meta-analysis results, we conducted sensitivity analysis by excluding the articles one by one in each step, and then obtained the meta-analysis results of the remaining studies. The results implied that the synthesized SMDs of NLR for DN (Figure 8A), NLR for DR (Figure 8B), as well as MPV for DN (Figure 8C), MPV for DR (Figure 8D) and PDW for DR (Figure 8E) did not change substantially when omitting any individual study, indicating that the results of meta-analysis were robust although significant heterogeneity existed amongst the included studies. Owing to the limitation of study number, sensitivity analysis was not applicable for the synthesized SMDs of the other data.

### Publication bias

**Figure 9:**
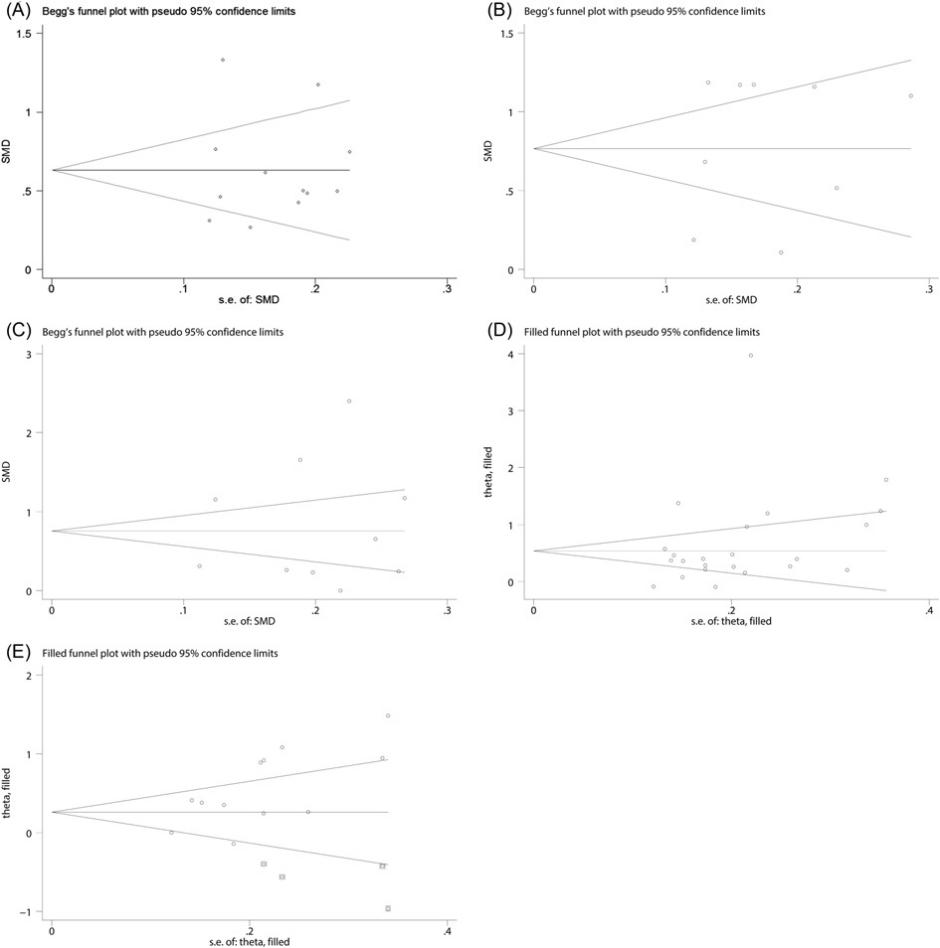
The funnel plots for the assessment of potential publication bias NLR for DN (**A**), NLR for DR (**B**), MPV for DR (**C**), MPV for DR (**D**), PDW for DR (**E**).

The assessment of publication bias of the included studies was performed by using Begg’s and Egger’s test. From the results of the publication bias test, we found that there was no evidence of publication bias for the pooled SMDs of NLR for DN (Begg’s test, *P*=0.244; Egger’s test, *P*=0.990) (Figure 9A) and for DR (Begg’s test, *P*=0.929; Egger’s test, *P*=0.588) (Figure 9B). Similarly, the publication bias for the pooled SMD of MPV for DN was not observed either (Begg’s test, *P*=0.371; Egger’s test, *P*=0.690) (Figure 9C).

Nevertheless, publication bias might exist amongst the eligible studies referring to MPV (Begg’s test, *P*=0.039; Egger’s test, *P*=0.148) (Figure 9D) and PDW (Begg’s tests, *P*=0.06; Egger’s tests, *P*=0.02) (Figure 9E) for DR. Consequently, we used meta-trim method to figure out the effect of publication bias on the dependability of the meta-analysis results. The results of meta-trim method showed that the adjusted SMDs of MPV and PDW were (0.68; 95% CI: 0.36–0.99) and (0.28; 95% CI: 0.018–0.540), respectively, indicating that the publication bias did not substantially affect the stability of the pooled results of the MPV and PDW for DR. In addition, the publication bias test was not applicable for the pooled SMD of PDW for DN owing to the limitation of the number of the eligible studies.

## Discussion

To our knowledge, this is the first meta-analysis focussing on the association of NLR, MPV, and PDW with DN and DR. In our meta-analysis, we found that NLR, MPV, and PDW was higher in patients with DR or DN. In addition, we also observed that MPV was strongly associated with the severity of DR, and NLR was closely related to the degree of DN.

White blood cells and their subtypes have been widely considered as inflammatory markers in various diseases including DM. NLR calculated by blood routine examination is readily available and inexpensive. It has better stability since it reflects the balance between the peripheral blood neutrophils and lymphocytes, which is less affected by various physiological and pathological status, as compared with independent neutrophils, lymphocytes, and total white blood cell count. In recent years, studies have reported that NLR might be a novel inflammatory marker, and there was a significant relationship of NLR to conventional markers of inflammation. For instance, several studies showed that there was a positive association of NLR with serum IL-6 and C-reactive protein [74,75]. Furthermore, it has been recognized that chronic inflammation has a significant role in the development and deterioration of DM-associated complications [76]. Several studies have reported that various inflammatory molecules such as adipokines, chemokines, adhesion molecules, and cytokines, could contribute to the development of DN [77]. A study showed that NLR, as a novel inflammation marker, is inversely associated with glomerular filtration rate (GFR) patients with chronic kidney disease [14]. Particularly, a 3-year follow-up study showed that NLR could serve as a predictor of worsening renal function in diabetes patients [78]. Additionally, numerous studies have shown that elevated NLR was associated with the presence of DN and MA [75,79–81]. In consistence with that, this meta-analysis confirmed that DM patients with DN, albuminuria, or MA have higher NLR, as compared with those without DN, albuminuria, or with MIA, which indicated that NLR plays an important role in the development and progression of DN.

It has also been demonstrated that chronic inflammation plays an important role in the development of DR with the help of various systemic and local (vitreous and aqueous fluids) inflammatory factors [82–85]. Neutrophils may contribute to the development and progression of microangiopathy and inflammation, when they adhere to the endothelial cell wall [86,87]. A study by Woo et al. [88] showed that neutrophil count in circulation was higher in patients with DR, and was closely associated with DR grades, indicating the considerable role of neutrophil-mediated inflammation in the development and progression of DR. Furthermore, several recent literatures have proposed that NLR, as a novel inflammation marker, was found to be higher in patients with DR and linked with the severity of DR [13,18,61,88,89]. However, in a study by Ciray et al. [79], NLR was not found to be associated with the pathogenesis of DR. In our meta-analysis, it was observed that NLR might be involved in the presence of DR, but not linked with the DR grade, indicating that NLR might only play a role in the early stage of DR. However, only four studies with the low number of cases were in place for the meta-analysis of the association between NLR and the DR grade. Therefore, the role of NLR in the later stage of DR remains controversial and further studies are in need to figure out this issue.

Albuminuria can lead to some changes in the levels of numerous biomarkers in blood circulation, which could be applied to diagnosis and the risk evaluation of progression of kidney diseases [90,91]. Several studies have shown that MPV and PDW values are higher in DM patients with DN when compared with those without DN. Similarly, our meta-analysis also demonstrated that DM patients with DN have increased level of MPV than those without DN. These results indicated that MPV and PDW values could be used for the diagnosis of DN. Furthermore, a few studies showed that the MPV values increased with DN stage proceeding, which suggested that MPV could be applied to determine the stage of DN and monitoring the progress of DN. Certainly, the exact causal association of MPV and PDW with DN need to be further explored, which will help physicians make full use of MPV and PDW in clinical practices.

It has been demonstrated that persistent presence of hyperglycemia, dyslipidemia, and insulin resistance could lead to endothelial and pericyte injury, which leaves DM patients in a ‘prothrombotic condition’ [92]. Furthermore, the morphologic and functional forms of platelets, which are related to enhanced platelet activity, were found to be increased in patients with DM [92]. Platelets with higher MPV value have more metabolic and enzymatic activities than the lower and produce more thromboxan-A_2_, b-thromboglobulin, and adhesion molecules releasing [19,20], which implicated that MPV might be connected with elevated thrombotic potential. In consistence with numerous studies focussing on the correlation of MPV with DR, this meta-analysis also showed that increased MPV was associated with the presence of DR and its severity. In addition, PDW is a parameter reflecting the variability of platelet size, and its high value indicated increased production of larger reticulated platelet, indicating that PDW value, similar to MPV, is closely linked with thrombotic potential. Consistently, from the results of meta-analysis, we found that higher PDW was related to the presence of DR, but there were no differences in PDW between patients with NPDR and PDR. Considering that only five studies were available for the meta-analysis of the relationship of PDW to the severity of DR, the small sample size might partly account for the negative association of PDW with the severity of DR. Thus, further studies are needed to further explore the correlation between PDW and the severity of DR.

When interpreting the results of the present study, several limitations should be taken into consideration. First, although random-effect model was applied, significant heterogeneity amongst studies was still observed. It might be attributed to differences in some characteristics of the included studies including age, ethnicity, diseases’ duration, and body mass index, in detecting instrument, in experimental designs and in quality of the included studies. Second, the number of included studies was insufficient for a persuasive meta-analysis of the association of NLR, MPV, and PDW with the DR grade. Third, the standard of the stage of DR/DN with T2DM patients was not uniform and may affect the results. Finally, this meta-analysis did not investigate the relationship of the NLR, MPV, and PDW to other factors that may influence the development of DR/DN, due to the lack of available data in the included studies.

In conclusion, this meta-analysis indicated that NLR MPV and PDW has a positive association with DN and DR. Furthermore, MPV is closely related to the severity of DR and DN. NLR, MPV, and PDW could be recommended as inexpensive diagnostic markers for DN and DR, and even antiplatelet agents might be effective to control the development of DN and DR. However, further high-quality clinical studies should be performed to investigate the relationship of NLR, MPV, and PDW to DN and DR.

## Abbreviations

CI: confidence interval
DM: diabetes mellitus
DN: diabetic nephropathy
DR: diabetic retinopathy
MA: macroalbuminuria
MIA: microalbuminuria
MPV: mean platelet volume
NLR: neutrophil to lymphocyte ratio
NOS: Newcastle–Ottawa scale
NPDR: non-proliferative DR
PDW: platelet distribution width
PDR: proliferative DR
SD: sufficient data
SMD: standardized mean difference
T2DM: type Ⅱ DM
